# When Science Replaces Religion: Science as a Secular Authority Bolsters Moral Sensitivity

**DOI:** 10.1371/journal.pone.0137499

**Published:** 2015-09-11

**Authors:** Onurcan Yilmaz, Hasan G. Bahçekapili

**Affiliations:** Department of Psychology, Dogus University, 34722, Acibadem, Istanbul, Turkey; University of Amsterdam, NETHERLANDS

## Abstract

Scientific and religious thinking compete with each other on several levels. For example, activating one generally weakens the other. Since priming religion is known to increase moral behaviour and moral sensitivity, priming science might be expected to have the opposite effect. However, it was recently demonstrated that, on the contrary, science priming increases moral sensitivity as well. The present set of studies sought to replicate this effect and test two explanations for it. Study 1 used a sentence unscrambling task for implicitly priming the concept of science but failed to replicate its effect on moral sensitivity, presumably due to a ceiling effect. Study 2 replicated the effect with a new measure of moral sensitivity. Study 3 tested whether science-related words create this effect by activating the idea of secular authority or by activating analytic thinking. It was demonstrated that words related to secular authority, but not words related to analytic thinking, produced a similar increase in moral sensitivity. Religiosity level of the participants did not influence this basic finding. The results are consistent with the hypothesis that science as a secular institution has overtaken some of the functions of religion in modern societies.

## Introduction

In parallel with the historical tension between science and religion, recent psychological studies have revealed a conflict in people’s minds between scientific and religious thinking. For example, exposing people to stimuli that prime science or analytic thinking leads to a reduction in religious faith [1]. In addition, intuitive thinkers are more likely to believe in God than analytic thinkers [2]. Similarly, the same study also found that when people are led to think analytically, they report less belief in God.

On the other hand, it is well known that exposing people to religious symbols leads them to more prosocial, and hence moral, behaviour [3]. Conversely, exposing people to scientific arguments to the effect that free will is an illusion makes them more likely to cheat in a subsequent task [4]. If one assumes the existence of very simple causal relationships among religious, scientific and moral thought, one would expect scientific thinking to lead to a weakening in morality. A recent study [5] revealed that, in contrast to that expectation, exposing people to stimuli that prime science leads to heightened sensitivity to punish a moral violation. Clearly, the relationship among scientific, religious and moral thought is more complex than initially assumed.

Implicit priming of both religious words (e.g., *divine*, *soul*, *God*) and secular words (e.g., *police*, *court*, *civil*) leads to increased prosocial behaviour [3]. Thus, one could argue that, at least in modern societies, secular institutions have overtaken religious institutions in their capacity to promote prosociality and living harmoniously as a moral society [6]. As an integral part of the secular society, science might be seen as representing secular authority and hence science priming can enhance prosociality by increasing moral sensitivity. Alternatively, science priming might activate analytic thinking and the notion of rationality and hence increase moral sensitivity. Preference for analytic thinking has been shown to influence wrongness judgments for moral violations [7] and inducing analytic thought has been shown to lead to more utilitarian moral judgments [8]. It is therefore not unreasonable to expect a similar influence of analytic priming on moral sensitivity.

The present research is designed to test these two alternative explanations of the effect reported by Ma-Kellams and Blascovich [5]. We first tried to replicate the original findings on a Turkish sample since the findings in question were obtained with relatively small samples. The replication attempt was further motivated by the general concern that psychological effects discovered in western samples may not be replicated in non-western samples (see [9, 10]) and by the recent failure of replication of traditional implicit religious priming effect on a Middle Eastern sample [11]. In the present case, while science priming increases moral sensitivity in a population like the US where science enjoys a real secular authority, the same may not be true in a presumably less secular and more religious population where science does not have the same privileged status. Study 1 and Study 2 attempted to replicate the original finding. Study 3 used either secular authority priming (hypothesized to activate the notions of justice) or analytic priming (hypothesized to activate rational thinking) to see whether either leads to an increase in moral sensitivity as compared to a neutral priming group.

## Study 1

### Methods

The ethics committee at Dogus University in Istanbul approved this study. All participants provided written informed consent and were fully debriefed at the end of the study.

#### Participants

Eighty three undergraduates (54 women, 27 men, two “no answer”) from Dogus University took part in Study 1 whose ages ranged between 18 and 28 (*M* = 21.05, *SD* = 1.82). All participants were native speakers of Turkish. They were randomly assigned to either the Science (*n* = 42) or the Neutral (*n* = 41) group. The majority of the participants were Muslim (*n* = 59). Of the remaining 24, 17 reported belief in God without being affiliated with a religion, three were atheists, and four did not answer. None of the participants could guess the hypotheses or the variables of the study during the debriefing given after the study.

#### Design and Procedure

The experiment was conducted in a classroom setting in several sessions, each involving 10–15 participants. The participants first took either science priming or neutral priming. They then read a date rape scenario (adapted from [5]) and indicated how wrong they viewed the action described on a 0–100 scale (going from “not wrong at all” to “extremely wrong”). The scenario involves a man and a woman who get close to each other after getting drunk and go to the woman’s apartment. After kissing, the woman does not want to go further but the man disregards her objections and consummates the relationship.

The manipulation involved a scrambled sentence task adapted from the one used by Ma-Kellams and Blascovich [5] (see also [12]). In both groups, the participants formed 10 sentences with four words each by taking out one of the given words and rearranging the remaining four. Five sentences in the science group contained the target words *logical*, *hypothesis*, *laboratory*, *scientist* and *theory*. Sentences in the neutral group did not contain any target words and did not form a coherent theme.

We also assessed the religiosity levels of the participants in order to see whether religiosity interacts with any of our variables. We asked the participants to rate to what extent they consider themselves as a religious person from 1 (not at all religious) to 7 (highly religious).

### Results and Discussion

Gender had neither a main effect on moral sensitivity nor significantly interacted with any other variable. Importantly, the priming manipulation did not have a significant main effect, *F*(1, 81) = 0.54, *p* = .464, *ηp²* = .007. Although mean wrongness rating in the Science group (*M* = 93.57, *SD* = 11.96; 95% CI [89.84, 97.30]) was higher than in the Neutral group (*M* = 91.34, *SD* = 15.49; 95% CI [86.45, 96.23]), it did not reach significance. When we controlled for religiosity as a covariate from 1 (not at all religious) to 7 (highly religious), the main effect of priming condition remained constant, *F* (1, 77) = 0.32, *p* = .576, *ηp²* = .004.

Although this result might indicate that the original findings are spurious, another possibility is that date rape is seen as obviously wrong by almost everyone in the Turkish culture and therefore we encountered a ceiling effect. This interpretation is supported by the observation that the majority of the participants in both groups (64% and 66%) gave the maximum wrongness score (100) for the moral violation in the scenario. We therefore tested the same effect with a milder scenario in the next study.

## Study 2

### Method

#### Participants

Fifty-eight undergraduates (49 women, 9 men) from Dogus University in Istanbul took part in Study 2 (mean age = 21.05, *SD* = 3.54). All participants were native speakers of Turkish. As in Study 1, they were randomly assigned to either the Science (*n* = 29) or the Neutral (*n* = 29) group. The majority of the participants were Muslim (*n* = 40). Of the remaining 18, seven reported belief in God without being affiliated with a religion, seven were atheists, and four did not answer. None of the participants could guess the hypotheses or the variables of the study during the debriefing given after the study.

#### Design and Procedure

The main procedural difference from Study 1 was the use of two different scenarios to measure moral sensitivity. One scenario represents the fairness/justice dimension of Haidt’s [13] Moral Foundations Theory and the other one represents the care/harm dimension. In the care/harm scenario, two people are driving fast to avoid being late for an important meeting. One of them realizes that they ran over and injured a cat. After thinking about it for a while, they decide not to take care of the cat and move on. In the fairness/justice scenario, one person buys a lottery ticket but leaves it unattended on a desk top. A friend of his gets hold of the ticket and learns that it won the big prize. After thinking about it for a while, he decides not to inform his friend and get the prize all by himself. The participants were asked to rate how wrong the action was in each scenario on a 0–100 scale as in Study 1. The order of the scenarios was counterbalanced.

### Results and Discussion

No analyses on the basis of gender were conducted since the number of men was too low. We first conducted a 2 (priming: science or neutral) x 2 (scenario type: care/harm or fairness/justice) mixed ANOVA (where the latter factor was within-subjects) on moral sensitivity. This analysis revealed no significant main effect of priming, *F*(1, 56) = 1.58, *p* = .215, *ηp²* = .*027*. However, there was a significant interaction between priming condition and scenario type, *F*(1, 56) = 8.48, *p* = .005, *ηp²* = .*132*. Since the effect of science priming on moral sensitivity is different for different dimensions of morality, we conducted separate one-way ANOVAs for different types of moral scenarios.

In the care/harm scenario, priming condition had a significant main effect on moral sensitivity, *F*(1, 56) = 8.00, *p* = .006, *ηp²* = .125. The Science priming group (*M* = 86.55, *SD* = 18.13; 95% CI [79.65, 93.45]) showed higher levels of moral sensitivity than the Neutral priming group (*M* = 71.72, *SD* = 21.64; 95% CI [63.49, 79.96]). When we controlled for religiosity, the results again revealed a significant main effect of priming in the care/harm scenario, *F*(1, 55) = 7.68, *p* = .008, *ηp²* = .123.

In the fairness/justice scenario, on the other hand, priming did not have a main effect on moral sensitivity, *F*(1, 56) = 0.31, *p* = .582, *ηp²* = .005. When we controlled for religiosity, the results remained constant, *F*(1, 55) = 0.51, *p* = .479, *ηp²* = .009.

The results indicate that science priming increases moral sensitivity in the care/harm scenario but not in the fairness/justice scenario. This suggests that being reminded of science influences only harm-related morality. In the next study, we investigated the possible mechanisms underlying this effect by testing two alternative hypotheses.

## Study 3

Study 3 tested whether science exerts its moral sensitivity boosting effect by activating analytic thinking or the idea of secular authority. We first conducted a pilot study to see whether science words indeed prime secular authority or analytic thinking. In this study, like in Studies 1 and 2, the participants were assigned either to the Science group (*n* = 18) or to the Neutral group (*n* = 17). They were then given a word stem completion task. In this task, participants were given two or three letters as the stem of a possible word and were asked to generate a word as quickly as possible that completes the given stem (e.g., SA_____ → SAMPLE or SANE). Five of the stems were the first two or three letters of words related to secular authority (*court*, *civil*, *judge*, *police*, *university*), five were the first two or three letters of words related to analytic thinking (*reason*, *analysis*, *logical*, *think*, *rational*), and five were neutral (*wood*, *paper*, *cry*, *bottle*, *handbag*). (The actual words used in the study were in Turkish since all participants were Turkish native speakers.) The results revealed that science priming indeed increased the accessibility of words related to secular authority: Participants in the Science group (*M* = 2.50, *SD* = 0.79; 95% CI [2.11, 2.89]) produced more secular authority words compared to the Neutral group (*M* = 1.82, *SD* = 0.81; 95% CI [1.41, 2.24]), *F*(1, 33) = 6.30, *p* = .017, *ηp²* = .160. The results were similar with respect to words related to analytic thinking: Participants in the Science group (*M* = 1.50, *SD* = 0.92; 95% CI [1.04, 1.96]) produced more analytic thinking words compared to the Neutral group (*M* = 0.65, *SD* = 0.61; 95% CI [0.34, 0.96]), *F*(1, 33) = 10.30, *p* = .003, *ηp²* = .238. These results suggest that priming science reminds of both secular authority and analytic thinking. The purpose of Study 3 was to determine through which of these paths science influenced moral judgments.

### Method

#### Participants

In Study 3, we estimated a medium effect (*f*) of .3, which required a total sample of 177 with 95% power of detecting any effect. We therefore determined the sample size to be 59 for each condition. One hundred and seventy seven undergraduates (146 women, 30 men, one “no answer”) from Yeditepe and Dogus University in Istanbul took part in Study 3 whose ages ranged between 18 and 35 (*M* = 22.41, *SD* = 6.09). All participants were native speakers of Turkish. They were randomly assigned to the Secular priming group (*n* = 59), the Analytic thinking priming group (*n* = 59) or the Neutral priming group (*n* = 59). The majority of the participants were Muslim (*n* = 131). Of the remaining 46, 28 reported belief in God without being affiliated with a religion, 15 were atheists, and three did not answer. One participant realized that the priming manipulation, which was presented by the experimenters as a linguistic ability test, had a different purpose, but still could not guess the real hypothesis of the study. Excluding this participant from the analyses did not alter the main findings. Thus, the analyses below include the entire sample.

#### Design and Procedure

The procedure was similar to that of Study 2. Only the care/harm scenario was used to measure moral sensitivity. The participants were randomly assigned to the Secular, Analytic or Neutral priming groups where they unscrambled 10 sentences. The five target words in the Secular group were *court*, *civil*, *judge*, *police*, and *contract* [3]. The five target words in the Analytic group were *ponder*, *analysis*, *logical*, *think*, and *rational* [1]. The words in the Neutral group did not form a coherent theme.

### Results and Discussion

**Fig 1:**
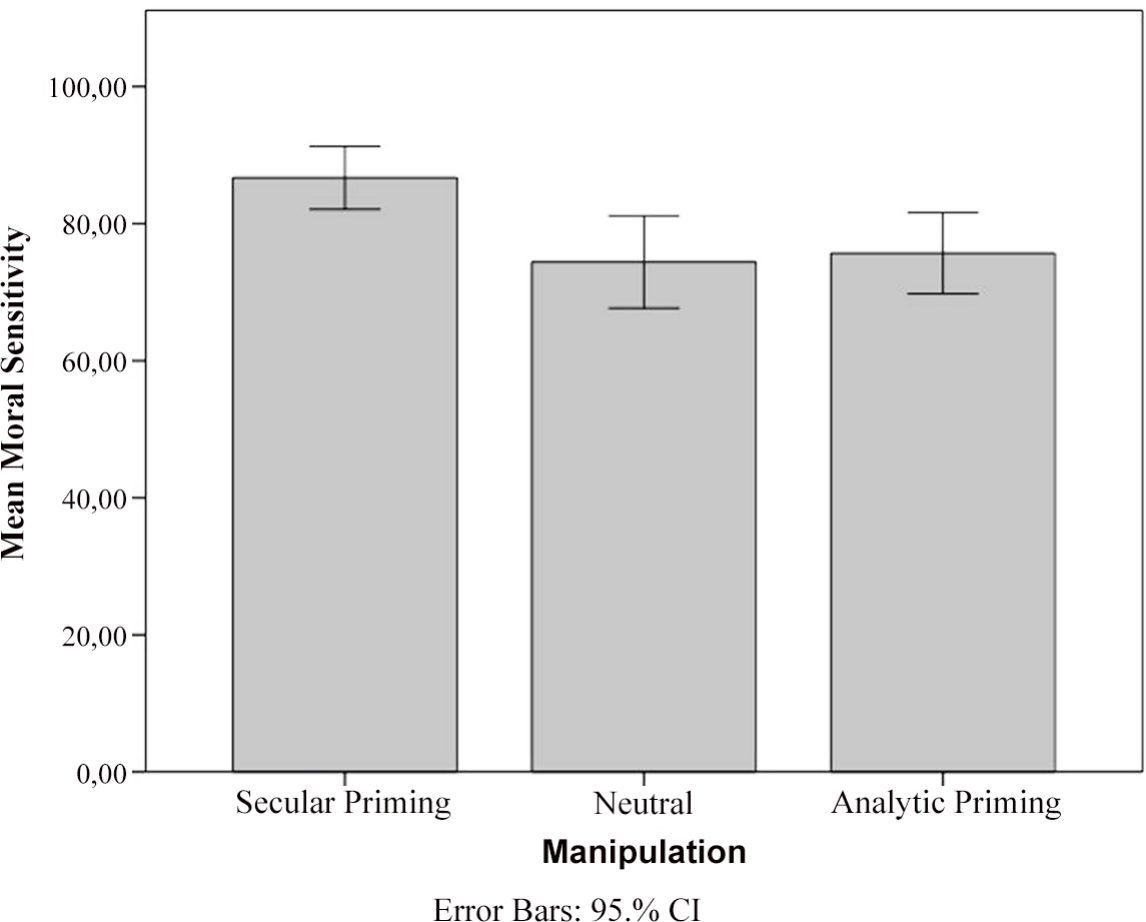
Moral Sensitivity Mean Scores for the Secular, Analytic, and Neutral Groups. Error bars represent 95% confidence intervals.

A two-way ANOVA revealed a significant main effect of gender, *F*(1, 170) = 4.23, *p* = .041, *ηp²* = .024: Females (*M* = 80.62, *SD* = 22.48) reported more moral sensitivity than males (*M* = 71.67, *SD* = 23.57). However, gender did not interact with the priming condition, *F*(1, 170) = 0.31, *p* = .732, *ηp²* = .004. A separate one-way ANOVA revealed a significant effect of priming condition on moral sensitivity, *F*(2, 174) = 5.40, *p* = .005, *ηp²* = .058. A Tukey HSD post hoc test demonstrated that the difference between the Secular (*M* = 86.69, *SD* = 17.66; 95% CI [82.09, 91.30] and the Neutral groups (*M* = 74.41, *SD* = 25.88; 95% CI [67.66, 81.15] was significant at *p* = .009 (*d* = 0.55) and the Secular and Analytic (*M* = 75.68, *SD* = 22.69; 95% CI [69.76, 81.59] groups was significant at *p* = .022 (*d* = 0.54), whereas the difference between the Analytic and the Neutral groups (*p* = .949; *d* = 0.05) was not significant (Fig 1). When we controlled for religiosity, the effect of priming remained constant, *F*(2, 173) = 5.17, *p* = .007, *ηp²* = .056. These results suggest that science priming exerts its boosting effect on moral sensitivity not by activating analytic thinking but by activating the idea of secular authority.

## General Discussion

This research replicated the effect of scientific thinking on moral sensitivity. In Study 1, we used the date rape scenario originally used by McKellams and Blascovich [5] but could not obtain a significant effect presumably because of a ceiling effect. In Study 2, we replicated the original finding by McKellams and Blascovich [5] with a care/harm scenario but not with a fairness/justice scenario. In Study 3, we demonstrated that the relation between science and moral sensitivity is due to the secular authority aspect of science and not due to its potential to activate analytic thinking.

In their Study 4, Ma-Kellams and Blascovich [5] found the effect of science priming on moral sensitivity in the domain of fairness/justice. One difference between the procedures of their study and our Study 2 is that they measured actual behaviour (money allocation in a dictator game) whereas we measured moral judgments on a hypothetical scenario (money allocation after a big win in lottery). It is possible that our scenario was not seen as sufficiently realistic and representative of real life moral decision making and therefore did not activate fairness concerns in the participants. It is also possible that the original findings by Ma-Kellams and Blascovich are spurious, a plausible interpretation given their small sample size and barely significant results (see [14]). It is therefore imperative to further test the effect of science priming on the fairness dimension of morality in future studies, preferably with more realistic scenarios (see [15] for examples of such scenarios).

The original contribution of the present research, the boosting effect of secular authority on moral judgment, together with the finding that science priming activates the idea of secular authority, is most plausibly explained by the hypothesis that science increases moral sensitivity through the activation of secular authority. This hypothesis is consistent with the previously demonstrated effect of science and secular authority on prosocial behaviour. For example, Ma-Kellams and Blascovich [5] demonstrated that science promotes prosocial behaviour as well as moral sensitivity. Similarly, Shariff and Norenzayan [3] demonstrated the influence of secular authority priming on prosocial behaviour. The power of religion to establish social harmony and to foster cooperation among genetically unrelated people is one of the most important factors behind human civilization [16–18]. However, in many modern societies where religiosity levels are at an all-time low, this historical role of religion has been largely overtaken by secular institutions [6]. In addition, there is evidence suggesting that people see religion and government as interchangeable sources of control and that decreased faith in one leads to increased faith in the other [19]. Thus, secular institutions motivate prosocial behaviour as much as religion [3]. The target words used in science priming in Study 2 (*hypothesis*, *laboratory*, *scientist*, *logical* and *theory*) might have primed the concept of science as a secular institution whose mission is to increase the welfare of society through technological progress (see [5]). Thus, our results are consistent with the idea that science is also a form of authority in modern societies able to motivate (at least some) moral sensitivity without moderation by religion.

On the other hand, religion is known to promote intergroup conflict as well as intragroup harmony [6]. Analogously, secular institutions can have both prosocial and antisocial effects. For example, in a classic study [20], the majority of the participants obeyed a scientific authority figure and (seemingly) shocked another participant at the highest possible level. This dual function of religious and secular institutions suggests an intriguing possibility: What religion or secular authority provides is not prosocial or antisocial behaviour per se but rather increased sensitivity for and conformity to (perceived) local norms. If this view is correct, being reminded of religion or secular authority should lead to antisocial behaviour if that is the behaviour consistent with the norm made salient by the relevant priming. The finding that synagogue-priming in Israeli settlers led to increased support for a suicide attack against Palestinians [21] seems to support this interpretation. The possible relation between science-priming and similar norm-compliant behaviour needs to be further investigated in future studies.

The conclusion drawn from this study might be made stronger by a mediation analysis showing the exact causal chain among science priming, accessibility of secular words and moral wrongness judgments. In addition, in Study 3 the absence of science priming is another limitation of our study. Therefore, future studies should evaluate the effect of science, secular authority and analytic thinking priming within the same design.

One further potential limitation of the research reported here is that it investigated moral norms only in the domains of care/harm and fairness/justice. Thus, another suggestion for further study is to explore the role of science, and of secular authority in general, in the other domains of morality (viz., authority/respect, ingroup/loyalty and purity/sanctity; [13]).

## Conclusion

Turkey is a country which has made its transition to secularism about 90 years ago, but where religious values are still strong. In this regard, it seems interesting that reminders of secular authority can function like reminders of religious monitoring on Turkish participants. It might be surmised that, rather than Western Europe or the rest of the Middle East, Turkey culturally resembles the US more since both religious and secular institutions have some degree of power in both. A recent indication of this resemblance is a poll on the public acceptance of evolutionary theory. Miller, Scott, and Okamoto [22] demonstrated that, in a survey of more than 20 countries, Turkey and the US were the two countries with the lowest degree of acceptance of evolutionary theory. One major predictor of disbelief in evolutionary theory turns out to be strong belief in a personal God and frequency of religious attendance. If our speculation about the cultural similarity of Turkey and the US is true, it might be interesting to try to replicate the present results in Scandinavian countries where religious authority has almost been completely replaced by secular authority, and also in some Middle Eastern countries where religion reigns supreme and secular institutions are still in a precarious situation.

## Supporting Information

S1 Dataset Full Dataset of Study 1. (XLSX)

S2 Dataset Full Dataset of Study 2. (XLSX)

S3 Dataset Full Dataset of Study 3. (XLSX)
